# Supplementary material for: Oat Polar Lipids Improve Cardiometabolic-Related Markers after Breakfast and a Subsequent Standardized Lunch: A Randomized Crossover Study in Healthy Young Adults
Source: Nutrients. 2021 Mar 18;13(3):988. doi: 10.3390/nu13030988 (PMC8003140; doi:10.3390/nu13030988)
Supplement: Supplementary file 1 [file nutrients-13-00988-s001.zip › Supplementary files/consort_flow_diagram_MMH.docx]

Analysed (n= 20*)

*Two subjects were excluded from statistical evaluation on appetite rating and plasma GIP due to missing appetite sensation scores and missing blood samples.

Figure 11: Flow diagram of the study progress

## Follow-Up

## Analysis

Lost to follow-up (give reasons) (n=0)

Discontinued intervention (give reasons) (n=0)

## Enrollment

Allocated to intervention (n=20)

 Received allocated intervention (n=20)

 Did not receive allocated intervention (give reasons) (n=0)

## Allocation

Randomized (n=20)

Excluded (n= 15)

  Not meeting inclusion criteria (n=5)

  Declined to participate (n=10)

  Other reasons (n=0)

Assessed for eligibility (n=35)
