# Supplementary material for: Oat Polar Lipids Improve Cardiometabolic-Related Markers after Breakfast and a Subsequent Standardized Lunch: A Randomized Crossover Study in Healthy Young Adults
Source: Nutrients. 2021 Mar 18;13(3):988. doi: 10.3390/nu13030988 (PMC8003140; doi:10.3390/nu13030988)
Supplement: Supplementary file 1 [file nutrients-13-00988-s001.zip › Supplementary files/Supplementary table_MMH.docx]

**Table *S*1.** Subjective appetite ratings after breakfast and lunch^1^.

| Test variables | NL | RSO |  | PLL |  | PLH |  |
| --- | --- | --- | --- | --- | --- | --- | --- |
|  |  |  | %∆^2^ |  | %∆^2^ |  | %∆^2^ |
| Satiety, fasting (mm) | 23.11±5.13 | 21.89±4.38 | -5.27 | 24.78±5.29 | 7.22 | 27.72±5.2 | 19.94 |
| Satiety, AUC 0-210 min (mm*min) | 9320±738 | 10137±687 | 8.76 | 9849±845 | 5.67 | 10125±800 | 8.63 |
| Satiety, AUC 210-330 min (mm*min) | 7259±444 | 7533±413 | 3.77 | 7286±449 | 0.37 | 7517±512 | 3.55 |
| Hunger, fasting (mm) | 60.17±5.48 | 62.44±5.6 | 3.77 | 57.67±5.6 | -4.15 | 62.44±5.22 | 3.77 |
| Hunger, AUC 0-210 min (mm*min) | 10522±796 | 10147±855 | -3.56 | 10090±829 | -4.10 | 9870±984 | -6.19 |
| Hunger, AUC 210-330 min (mm*min) | 4510±513 | 4595±576 | 1.88 | 4461±556 | -1.08 | 4216±488 | -6.51 |
| Desire to eat, fasting (mm) | 67.33±5.16 | 65.28±6.94 | -3.04 | 62.78±6.06 | -6.75 | 65.28±9.94 | -3.04 |
| Desire to eat, AUC 0-210 min (mm*min) | 12130±1019 | 11583±1083 | -4.50 | 11041±1057 | -8.97 | 10322±1074 | -14.90 |
| Desire to eat, AUC 210-330 min (mm*min) | 5298±652 | 5556±635 | 4.86 | 5236±690 | -1.17 | 4933±553 | -6.88 |

^1^Data are presented as means ± SEM, n=18 healthy subjects. ^2^The percentage change is calculated as the difference from the NL. NL, oat preparation without added lipids; RSO, oat preparation added with rapeseed oil; PLL, oat preparation with low concentration of polar lipids; PLH, oat preparation with high concentration of polar lipids; AUC, area under curve.
